# KIF22 regulates mitosis and proliferation of chondrocyte cells

**DOI:** 10.1016/j.isci.2024.110151

**Published:** 2024-05-31

**Authors:** Hiroka Kawaue, Takuma Matsubara, Kenichi Nagano, Aoi Ikedo, Thira Rojasawasthien, Anna Yoshimura, Chihiro Nakatomi, Yuuki Imai, Yoshimitsu Kakuta, William N. Addison, Shoichiro Kokabu

**Affiliations:** 1 Division of Molecular Signaling and Biochemistry, Department of Health Improvement, Kyushu Dental University, Kitakyushu, Fukuoka 803-8580, Japan; 2 Department of Oral Pathology, Institute of Biomedical Sciences, Nagasaki University, Nagasaki, Nagasaki 852-8588, Japan; 3 Division of Integrative Pathophysiology, Proteo-Science Center, Ehime University, Toon, Ehime 791-0295, Japan; 4 Division of Physiology, Department of Health Improvement, Kyushu Dental University, Manazuru, Kitakyushu, Fukuoka 803-8580, Japan; 5 Laboratory of Structural Biology, Graduate School of Systems Life Sciences, Kyushu University, Fukuoka, Fukuoka 819-0395, Japan

**Keywords:** cell biology, Molecular biology

## Abstract

Point mutations in *KIF22* have been linked to spondyloepimetaphyseal dysplasia with joint laxity, type 2 (SEMDJL2). Skeletal features of SEMDJL2 include short stature and joint laxity. Mechanisms underlying these limb abnormalities are unknown. Here in this manuscript, we have investigated the function of KIF22 in chondrocytes. Quantitative PCR and immunostaining revealed that *Kif22* was highly expressed in proliferating-zone growth-plate chondrocytes. *Kif22* knockdown resulted in defective mitotic spindle formation and reduced cell proliferation. Forced expression of SEMDJL-associated mutant *Kif22* constructs likewise induced abnormal mitotic spindle morphology and reduced proliferation. Mice expressing a KIF22 truncation mutant had shorter growth plates and shorter tibial bones compared to wild-type mice. These results suggest that KIF22 regulates mitotic spindle formation in proliferating chondrocytes thereby linking the stunted longitudinal bone growth observed in SEMDJL2 to failures of chondrocyte division.

## Highlights

• KIF22 facilitates chondrocyte proliferation by regulating mitosis
• Expression of KIF22 SEMDJL2 mutants cause defective cell proliferation *in vitro*
• KIF22 truncation mutant mice exhibit growth plate abnormalities

## Graphical abstract

**Figure:**
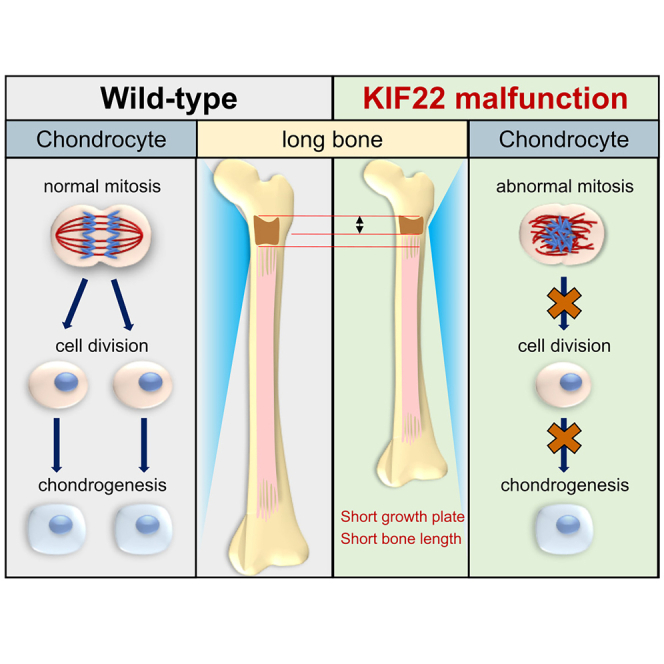

## Introduction

Spondyloepimetaphyseal dysplasia with joint laxity, type 2 (SEMDJL2) is an autosomal inherited disorder characterized by short stature, short limb bones, central facial hypoplasia, progressive knee joint malalignment, and generalized laxity of the ligaments.^1^^,^^2^ Whole-exome sequencing analysis links point mutations at Pro148, Arg149, and Arg232 of a kinesin family protein, KIF22, to SEMDJL2.^1^^,^^2^ Short stature and facial hypoplasia are also observed in chromosome 16p11.2p12.2 microdeletion syndrome, wherein the chromosomal region containing *KIF22* is also deleted.^3^^,^^4^^,^^5^ The clinical features of these disorders suggest an importance of KIF22 in skeletal development.

The growth plate, a cartilaginous tissue between the epiphysis and metaphysis of long bones plays a critical role in bone growth and elongation.^6^^,^^7^^,^^8^ The growth plate is divided into a resting zone, proliferating zone, and hypertrophic zone.^9^^,^^10^^,^^11^^,^^12^ Quiescent skeletal stem cells reside in the resting zone. These cells differentiate into chondrocytes and undergo cell division in the proliferating zone. Chondrocytes in the proliferating zone are flat and aligned in columns. They secrete a cartilaginous extracellular matrix (ECM) containing type 2 collagen and aggrecan.^10^^,^^13^ Toward the hypertrophic zone, the chondrocytes become progressively larger and rounder. Hypertrophic chondrocytes calcify as they approach the diaphysis and replace the cartilaginous matrix with bone tissue.^10^ Thus, chondrocyte cell division in the proliferating zone provides a supply of cells vital for elongation of the growth plate and long bones.

As cells proliferate, they progress through cell-cycle event phases described as G1, S, G2, and M.^14^^,^^15^ G1 and G2 are preparatory phases for events that occur in S and M phases. During the S phase, DNA is replicated.^15^ M phase is further divided into four phases: prophase, prometaphase, metaphase, and anaphase. Prophase consists of nuclear membrane loss, DNA aggregation (chromosome formation), and migration of the separated centrosome to the poles. From prometaphase to metaphase, chromosomes are localized at the equatorial plane, and bind to microtubule spindle fibers extending from the poles. During anaphase, chromosomes are pulled by the spindle fibers to each pole. Finally, a new nuclear membrane is formed, and the cytoplasm segregated to complete cell division.^16^^,^^17^^,^^18^^,^^19^ KIF22 is likely to be associated with M phase events such as spindle formation since KIF22 expression is upregulated at the beginning of M phase and downregulated immediately at the end of M phase.^20^^,^^21^^,^^22^^,^^23^

KIF22, like other kinesin family proteins, transports molecules along microtubules. KIF22 consists of a motor domain, a DNA-binding domain (DB), and a microtubule-binding (MTB) domain.^20^ The motor domain contains ATPase activity and hydrolyzes ATP to provide energy for movement of KIF22 toward the plus end of microtubules.^22^^,^^24^^,^^25^ During prometaphase, KIF22 guides DNA to the equatorial plate. During metaphase, KIF22 helps transport DNA from the equatorial plate toward the poles.^26^ In *Kif22* knockout mouse embryos chromosome cohesion and partitioning do not occur normally, and cell division frequently stops early in embryogenesis. Thus, viable mice are rarely born. On rare occasions where cell division proceeds past the morula stage, mitosis is normal thereafter and viable pups grow into healthy adult animals.^27^ The requirement for KIF22 in cell division among different cell types is unknown. Proliferating cells in mouse growth plate cartilage highly express *Kif22* suggesting that KIF22 may be involved in the longitudinal growth of bone by regulating proliferation.^2^ Although functional studies examining the role of KIF22 in proliferation have been conducted in cancer cells,^28^^,^^29^ the role of Kif22 in chondrocytes remains unknown.

In this study, we found that KIF22 was highly expressed in the proliferating zone of cartilage. We observe that in chondrocyte cells, KIF22 is necessary for normal chromosome segregation, mitotic-spindle fiber formation, and cell division. Mutant mice expressing a truncated KIF22 protein had shortened growth-plate cartilage and reduced long-bone length. Furthermore, overexpression of a KIF22 SEMDJL2 mutant variant (Pro143Leu) resulted in abnormal spindle fiber formation and reduced cell division of ATDC5 chondrocyte cells. These results suggest that KIF22 facilitates long-bone growth by regulating chondrocyte cell division. Skeletal defects in SEMDJL2 may potentially be a result of abnormal chondrocyte proliferation.

## Results

### KIF22 is expressed in growth plate cartilage

**Figure 1:**
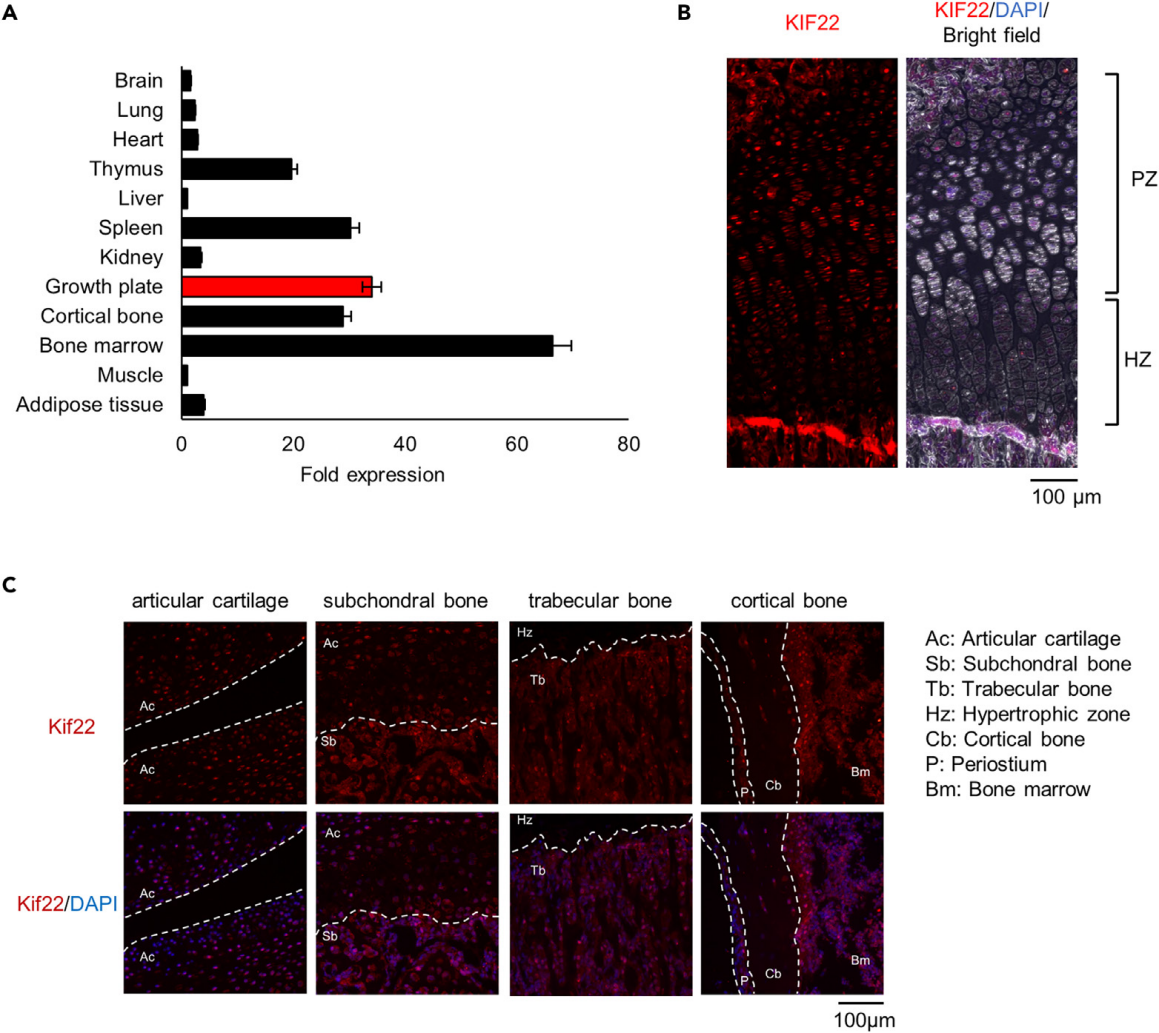
KIF22 is highly expressed in proliferating-zone chondrocytes of the growth plate (A) qPCR analysis of *Kif22* mRNA in various organs and tissues of 5-week-old mice. Data are represented as mean ± SD; *n* = 3. Other statical data were shown in Table S1. (B) Immunofluorescent staining of KIF22 at the growth plate of tibia from 2-week-old mice. The proliferating zone (PZ) and hypertrophic zone (HZ) of the growth plate are indicated. Scale bar, 100 μm. (C) Immunofluorescent staining of KIF22 at the growth plate of tibia from 2-week-old mice. Images of articular cartilage, subarticular subchondral bone, trabecular bone below under the growth plate, and cortical bone.

We first examined the distribution of *Kif22* mRNA expression across various mouse tissues. Real-time qPCR (qPCR) analysis showed that *Kif22* was highly abundant in growth plate and bone marrow (Figure 1A). To determine KIF22 localization in bone tissues, sections of tibia growth plate from 2-week-old mice were stained by immunohistochemistry with KIF22 antibodies. As shown in Figure 1B, KIF22 protein was detected in chondrocytes at the proliferative zone. KIF22 protein was also detected in cells within subchondral bone and in cells present in the marrow space (Figure 1C). These observations are consistent with the qPCR data. KIF22 expression in chondrocytes suggests that KIF22 may be important for the specialized function of proliferative-zone chondrocytes.

### KIF22 is essential for cell proliferation and subsequent matrix synthesis

**Figure 2:**
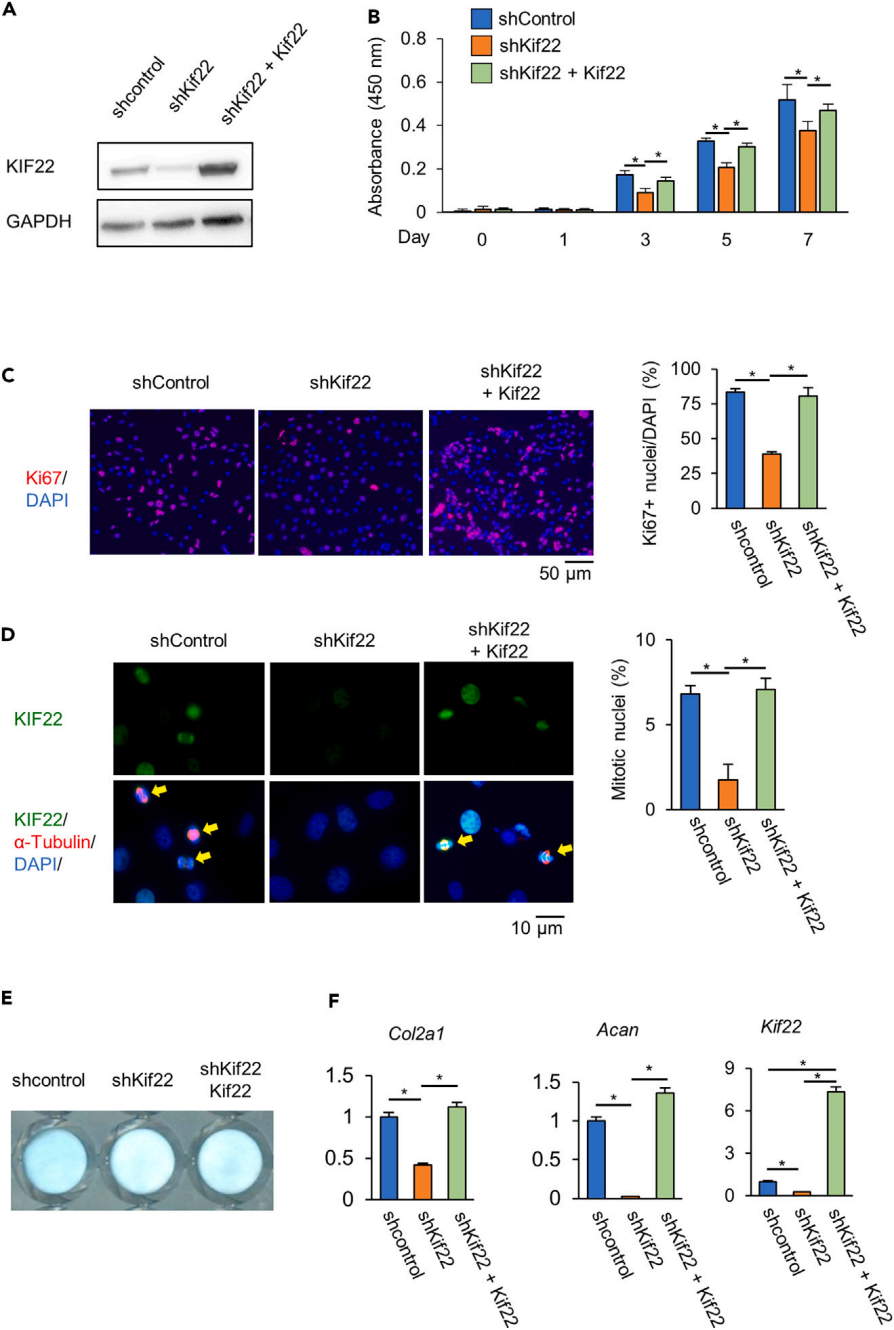
KIF22 is essential for cell proliferation in ATDC5 cells ATDC5 cells were infected with control shRNA adenovirus (shcontrol), Kif22 shRNA adenovirus (shKif22), and/or Myc-tagged-Kif22 overexpression adenovirus (Kif22). (A) Kif22 expression was examined by western blotting analysis. Uncropped image was shown in Figure S1. (B) ATDC5 cells were plated at 3,000 cells/cm^2^ in 96-well plates. CCK8 cell viability assay showing proliferation of ATDC5 cells. Data are represented as mean ± SD; *n* = 4. ∗; *p* < 0.05. Other statical data were shown in Table S1. (C) Ki67 immunofluorescence staining and quantification 48 h after infection. Scale bar, 50 μm. Data are represented as mean ± SD; *n* = 3. ∗; *p* < 0.05. Other statical data were shown in Table S1. (D) KIF22 and tubulin immunostaining 24 h after infection and cell synchronization. Nuclei were counterstained with DAPI. Mitotic nuclei (arrows) were quantified. Scale bar, 10 μm. Data are represented as mean ± SD; *n* = 4. ∗; *p* < 0.05. Other statical data were shown in Table S1. (E and F) Alcian blue staining (E) and qPCR analysis (F), 7 days after induction of differentiation. Data are represented as mean ± SD; *n* = 4. ∗; *p* < 0.05. Other statical data were shown in Table S1. More information is available at Figure S1.

To examine the role of KIF22 in chondrocytes, we knocked down *Kif22* in ATDC5 chondrogenic cells using shRNA. A cell viability assay showed that the growth rate of *Kif22* knockdown cells was significantly reduced compared to control cells (Figures 2A, 2B, and S1). *Kif22* knockdown did not affect apoptosis as measured by TUNEL or cleaved caspase 3 staining (Figure S2). In contrast, immunostaining for Ki67, a marker of cell proliferation, revealed that *Kif22* knockdown decreased the number of Ki67-positive cells (Figure 2C). Furthermore, the number of mitotic cells in shKif22 cultures was also reduced (Figure 2D).

ATDC5 cells treated with insulin-transferrin-selenium (ITS) proliferate and secrete an extracellular matrix containing type II collagen and aggrecan, similar to that of proliferating zone chondrocytes.^30^^,^^31^ To determine whether KIF22 affects extracellular matrix production, Alcian blue staining for proteoglycans was performed on day 7 of differentiated ATDC5 cultures. Alcian blue stain intensity was reduced in *Kif22* knockdown cells compared to controls (Figure 2E). Furthermore, mRNA levels of collagen type II alpha 1 (*Col2a1*) and aggrecan (*Acan*) were significantly reduced by *Kif22* knockdown (Figure 2F). The reduction of cell proliferation and matrix synthesis by *Kif22* knock down was rescued by forced expression of KIF22 (Figure 2). These results suggest that loss of KIF22 affects the proliferation and subsequent matrix synthesis of ATDC5 by interfering with mitosis.

### *Kif22* mutant mice have a wider joint space and a shortened growth plate

**Figure 3:**
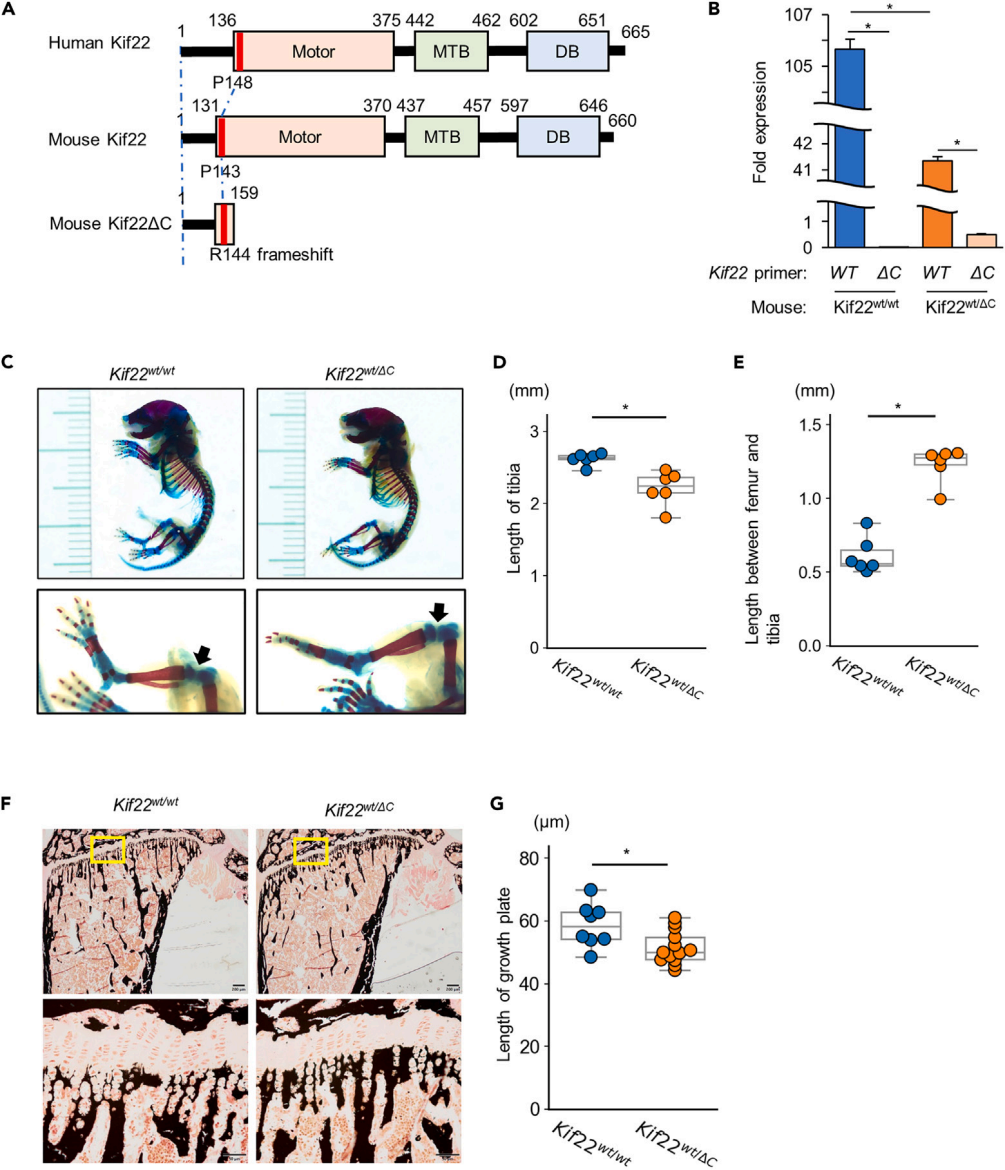
*Kif22* mutant mice have a wider joint space and shorter growth plate (A) Schematic diagram of human KIF22, mouse KIF22 and the mouse KIF22 R144fs∗16 truncated C-terminal mutant (Kif22ΔC). Motor; motor domain, MTB; microtubule binding domain, DB; DNA binding domain. The red lines indicate the location of SEMDJL2 patient mutations and the corresponding amino acid in mouse. (B) Kif22 wild-type (WT) and Kif22 ΔC (ΔC) mRNA expression in primary chondrocytes from Kif22^w/w^ and Kif22^wt/ΔC^ mice were determined by qPCR using allele specific primers. Data are represented as mean ± SD; *n* = 3. ∗; *p* < 0.05. Other statical data were shown in Table S1. (C) Alcian blue and Alizarin red staining of skeletal preparations of 1-day-old *Kif22*^*w/w*^ and *Kif22*^*wt/ΔC*^ mice. Representative images of *Kif22*^*w/w*^ and *Kif22*^*wt/ΔC*^ was shown. Arrow indicates joint space between proximal tibia and distal femur. Scale increments are 1 mm. (D) Measurement of tibia length and (E) joint space from the skeletal preparations shown in (C). Each data are represented by a dot. The statistical data are also represented by box-and-whisker diagrams. ∗; *p* < 0.05. Other statical data were shown in Table S1. (F) von Kossa stained sections of proximal tibia from 8-week-old *Kif22*^*w/w*^ or *Kif22*^*wt/ΔC*^ mice. Representative images of *Kif22*^*w/w*^ and *Kif22*^*wt/ΔC*^ are shown. Yellow boxed region is shown magnified in lower panel. Low and high magnification scales are 200 μm and 50 μm, respectively. (G) Measurement of growth plate thickness in samples shown in (F). Each data are represented by a dot. The statistical data are also represented by box-and-whisker diagrams. ∗; *p* < 0.05. Other statical data were shown in Table S1.

**Figure 4:**
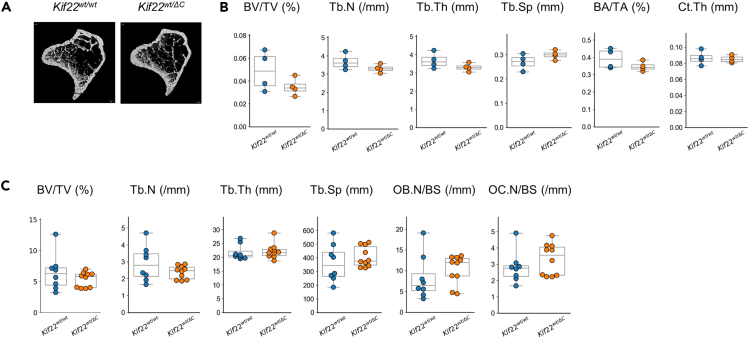
MicroCT and histomorphometric analysis of *Kif22* mutant mice MicroCT analysis (A and B) and histomorphometric analysis (C) of tibia from 8-week-old mice was performed. (A) Representative microCT image of littermates. Parameters; Bone volume/Tissue volume (BV/TV), Trabecular number (Tb.N), Trabecular thickness (Tb.Th), Trabecular separation (Tb.Sp), Cortical bone area/Tissue area (BA/TA), Cortical thickness (Ct.th), Osteoblast number/Bone surface (OB.N/BS), Osteoclast number/Bone surface (OC.N/BS). Each data are represented by a dot. The statistical data are also represented by box-and-whisker diagrams. Other statical data were shown in Table S1.

To examine the *in vivo* function of KIF22 on growth plate formation, we generated mutant mice with a truncated KIF22 C-terminus (KIF22ΔC). The resulting truncated protein lacks a complete motor domain and is also missing the MTB and DB domains (Figure 3A). Attempts to obtain homozygous mutants were unsuccessful, suggesting that homozygosity leads to embryonic lethality. This confirms the previously reported homozygous lethality of Kif22 mutant mice lacking Exon 3 to Exon 11.^27^ Mice heterozygous for the Kif22 mutant allele (*Kif22*^*wt/ΔC*^) were viable and fertile. Using allele-specific primers we found that the expression level of the wild-type allele was reduced by half in the chondrocytes from *Kif22*^*wt/ΔC*^ mice. Expression of the mutant *Kif22ΔC* allele mRNA was significantly lower than wild-type *Kif22* mRNA (Figure 3B). Examination of skeletal preps at P1 showed that *Kif22*^*wt/ΔC*^ mice had shorter tibia than wild-type mice (Figures 3C and 3D). Furthermore, the joint space between femur and tibia were longer in mutant mice than in wild-type mice (Figures 3C and 3E). To further investigate the shorter bone length in mutant mice, we examined the growth plates of 8-week-old mouse tibia and found that mutant mice had significantly shorter growth plates (Figures 3F and 3G). Quantitative analysis of trabecular and cortical bone parameters in tibia by microCT analysis revealed no major differences between wild-type and *Kif22*^*wt/ΔC*^ mice (Figure 4).

### Kif22 mutations disturb spindle fiber formation during mitosis

**Figure 5:**
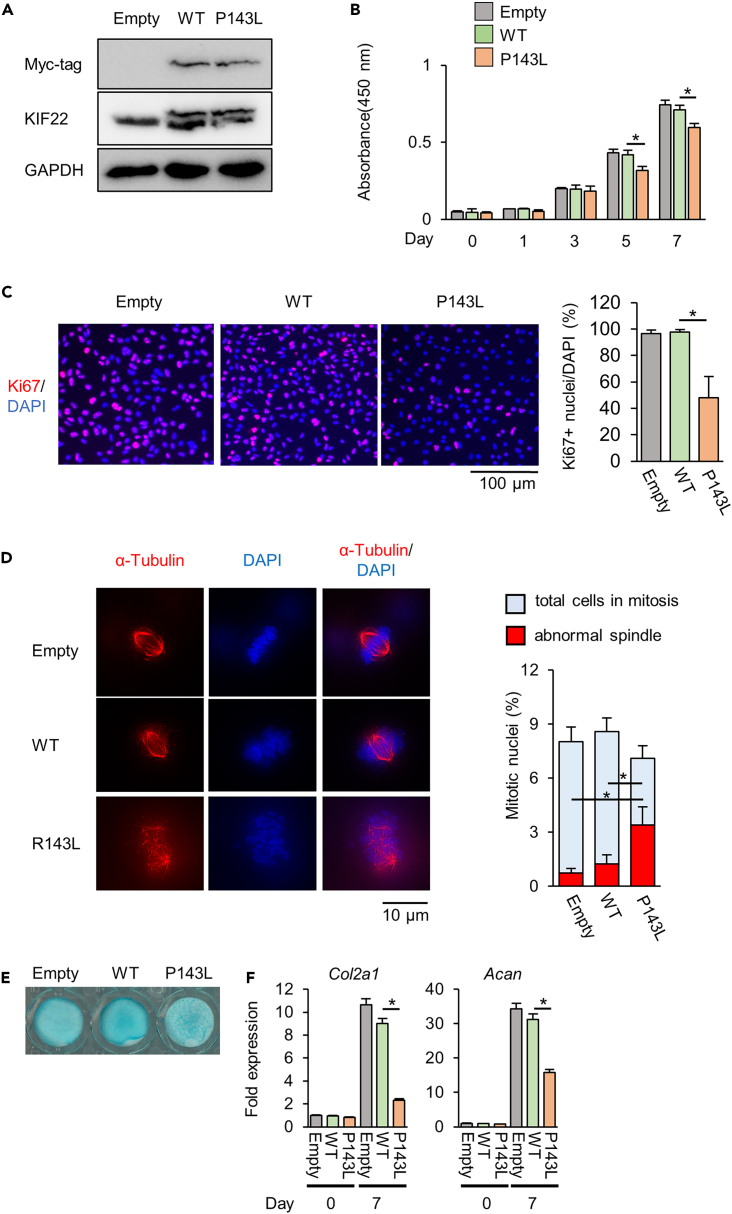
Kif22 P143L mutation disturbs spindle fiber formation during mitosis ATDC5 stably expressing empty pcDNA3.1 plasmid (empty), myc-tagged wild-type Kif22 (WT) and Kif22 P143L (P143L) were generated. (A) Expression of endogenous and exogenous KIF22 proteins were determined by western blotting analysis. Uncropped image was shown in Figure S3. (B) CCK8 cell viability assay of ATDC5 cells. Data are represented as mean ± SD; *n* = 4. ∗; *p* < 0.05. Other statical data were shown in Table S1. (C) Ki67 immunofluorescence staining and quantification of WT and P143L at 24 h after seeding. Scale bar, 50 μm. Data are represented as mean ± SD; *n* = 4. ∗; *p* < 0.05. Other statical data were shown in Table S1. (D) Tubulin immunostaining of WT and P143L cells after cell synchronization. Nuclei were counterstained with DAPI. Total number of mitotic nuclei and abnormal spindles were quantified. Scale bar, 10 μm. (Data are represented as mean ± SD; *n* = 3. ∗; *p* < 0.05. Other statical data were shown in Table S1. (E) Alcian blue staining and (F) qPCR analysis of the indicated cells 7 days after induction of differentiation. Data are represented as mean ± SD; *n* = 3. ∗; *p* < 0.05. Other statical data were shown in Table S1. More information is available at Figure S2.

**Figure 6:**
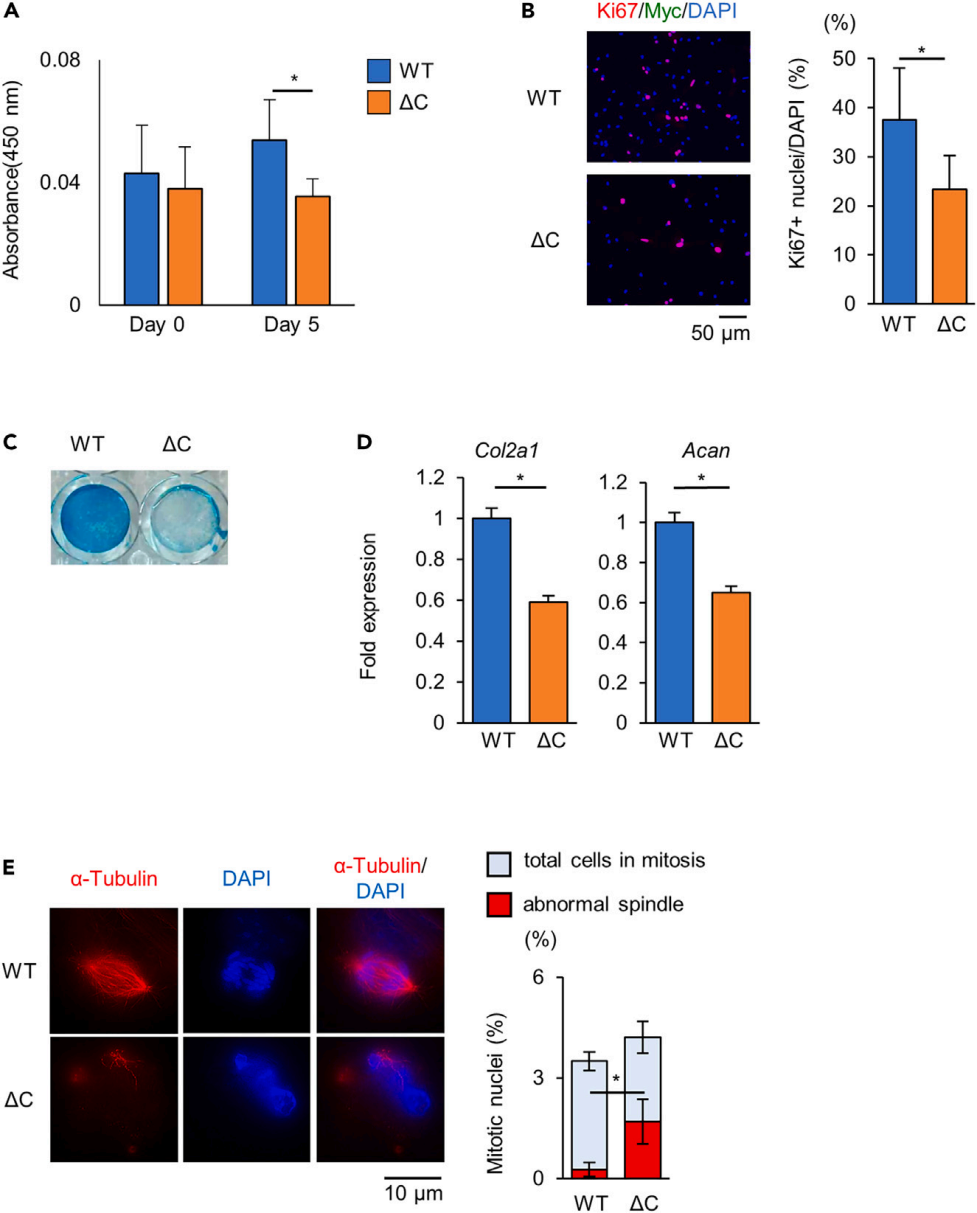
Spindle fiber formation and cell mitosis was disturbed in *Kif22*^*wt/ΔC*^ chondrocytes Chondrocytes were harvested from costal cartilage of P2 mice. (A) CCK-8 cell viability assay showing proliferation. Data are represented as mean ± SD; *n* = 4. ∗; *p* < 0.05. Other statical data were shown in Table S1. (B) Ki67 immunofluorescence staining and quantification of *Kif22*^*w/w*^ (WT) or *Kif22*^*wt/ΔC*^ (ΔC) primary chondrocytes 24 h after plating. Scale bar, 50 μm. Data are represented as mean ± SD; *n* = 3. ∗; *p* < 0.05. Other statical data were shown in Table S1. (C) Alcian blue staining and (D) qPCR analysis of WT or Kif22ΔC cells 7 days after plated with chondrogenic media. Data are represented as mean ± SD; *n* = 3. ∗; *p* < 0.05. Other statical data were shown in Table S1. (E) Tubulin immunostaining of WT or Kif22ΔC cells 24 h after plating and cell synchronization. Nuclei were counterstained with DAPI. Total number of mitotic nuclei and abnormal spindles were quantified. Scale bar, 10 μm. Data are represented as mean ± SD; *n* = 3. ∗; *p* < 0.05. Other statical data were shown in Table S1. More information is available at Figure S3.

We next addressed whether SEMDJL2-associated mutant variants of KIF22 affect chondrogenesis. KIF22 P143L (equivalent to P148L in human KIF22), a KIF22 missense mutation occurring in SEMDJL2, was stably expressed in ATDC5 cells (Figures 5A and S3). KIF22 P143L ATDC5 cells had decreased cell proliferation with no effect on apoptosis (Figures 5B, 5C, and S4A). The percentage of cells with disturbed spindle fiber formation and abnormal chromosome aggregation was increased in KIF22 P143L ATDC5 cells (Figures 5D and S4B). In addition, KIF22 P143L mutant ATDC5 cells had reduced expression of *Col2a1* and *Acan* (Figures 5E and 5F). We next examined primary chondrocytes from costal cartilage of newborn wild-type and Kif22 mutant mice. CCK8 cell viability assays showed that chondrocytes from *Kif22*^*wt/ΔC*^ mice had a reduced rate of cell proliferation compared to wild-type cells (Figure 6A). Furthermore, the number of Ki67 positive cells was also reduced in chondrocytes from *Kif22*^*wt/ΔC*^ mice (Figure 6B). These observations were similar to and consistent with the effects of *Kif22* knockdown (Figure 2). Following differentiation, Alcian blue staining (Figure 6C) and expression of *Col2a1* and *Acan* (Figure 6D) were also reduced in chondrocytes from *Kif22*^*wt/ΔC*^ mice. Examination of mitosis revealed that abnormal mitotic spindle formation was significantly increased in chondrocytes from *Kif22*^*wt/ΔC*^ mice (Figures 6E and S5). These data suggest that mutation of Kif22 prevents mitosis by disturbing spindle formation.

## Discussion

Knockdown of KIF22 inhibited cell division and reduced the chondrogenic differentiation potential of ATDC5 cells. Since KIF22 is highly expressed within proliferative zone cells of the growth plate, KIF22 may be involved in chondrocyte proliferation and bone elongation. Consistent with this, *Kif22*^*wt/ΔC*^ mice had shorter growth plates and shorter limb bones than wild-type mice. Similarly, SEMDJL2 disease variants, P143L and R144Q, disrupted spindle fiber formation and suppressed division of ATDC5 cells. Taken together, the SEMDJL2 features of short stature and shortened limbs may be the result of abnormal chondrocyte cell division caused by mutations in KIF22.

In ATDC5, knockdown of *Kif22* resulted in defective mitotic spindle formation and reduced cell division. In addition to cellular transport along microtubules, kinesins also regulate microtubule morphogenesis, length and curvature by modulating tubulin binding activity and degradation.^32^ Similar to our observations, *Kif22* knockdown in HeLa cells has been reported to result in abnormal spindle formation, chromosome compaction, and cell division defects.^26^^,^^27^^,^^32^^,^^33^ Based on these observations, the authors suggested that KIF22 regulates directional determination and extension of microtubule polymerization. We speculate that SEMDJL2 mutants are unable to bind tubulin because they lack the ATPase activity required for a conformational change of the motor domain to bind microtubule for spindle formation.

Kif22 knockdown in ATDC5 led to reduced proliferation and differentiation. Forced expression of KIF22 in knockdown cells could rescue this phenotype. However, overexpression of Kif22 in normal ATDC5 cells did not increase proliferation or differentiation. Therefore, although KIF22 may be essential for cell proliferation, the basal level of KIF22 is sufficient for normal cell function.

We observed that growth plates in *Kif22*^*wt/ΔC*^ mice were shorter than in wild-type mice. Defective chondrogenesis is often associated with defective endochondral ossification. Interestingly, microCT and bone morphometry analysis of *Kif22*^*wt/ΔC*^ mice at 8-weeks-old revealed no statistically significant differences in trabecular and cortical bone parameters. It is certainly possible that alterations in bone mass may be transient or age-dependent. Skeletal defects in SEMDJL2 patients become progressively worse with age.^34^ Epiphyseal dysplasia is more pronounced beyond 7-year of age.^34^ The relationship between bone mass and SEMDJL2 is currently unknown.^1^^,^^2^^,^^34^ Therefore, further analysis of *Kif22*^*wt/ΔC*^ mice at various ages may help to better understand the role of Kif22 in bone homeostasis.

KIF22’s role as a mitogenic factor also links it to cancer cell proliferation. KIF22 is highly expressed in breast, ovarian, lung, and prostate cancers wherein it is associated with a worse prognosis.^35^^,^^36^ In cancer cells, inhibition of KIF22 leads to a G2/M phase accumulation and disrupted cell cycle progression.^34^ Mechanistically, it was observed that in cancer cells independent of KIF22’s microtube-dependent function, KIF22 binds to and transcriptionally represses the promoter region of Cdc25C, an inhibitor of mitosis termination.^35^ This indicates that KIF22 may also function as a transcriptional regulator via its DNA-binding activity. In fact, SEMDJL2 mutants of KIF22 may affect the transcriptional activity of SOX9 (Figure S6). Further studies will be required to determine the details of KIF22’s function as a transcriptional regulator in chondrocytes.

In summary, this study revealed that KIF22 is highly expressed in growth plate chondrocytes and regulates chondrocyte cell division. *Kif22*^*wt/ΔC*^ mice have shorter growth plates. Furthermore, KIF22 mutation inhibits mitotic spindle formation, and prevents chondrocyte proliferation. This could potentially lead to the shortened limbs and short stature of SEMDJL2.

### Limitations of this study

Some potential limitations should be taken into consideration when interpreting this study. Firstly, the *in vivo* mouse model investigated was a frameshift truncation mutant. We cannot completely exclude the possibility that the phenotype is caused by an unknown function of the truncated protein. Secondly, the KIF22 mutation in the mouse model is globally expressed and not chondrocyte specific. Systemic effects derived from crosstalk between non-chondrogenic cells and chondrocyte cells could contribute to the skeletal phenotypes observed. Although the *ex vivo* primary chondrocyte data strongly points toward a cell-autonomous chondrocyte phenotype, we cannot completely rule out the contribution of non-chondrogenic cells to the growth plate phenotype. Thirdly, several mechanistic conclusions are based on KIF22 mutant overexpression in cells which have a background level of basal wild-type KIF22 expression. Although this approach perhaps mimics the heterozygous nature of human SEMDJL2 patients, it may not necessarily distinguish between dominant-negative effects and the effects of excess KIF22 protein.

## STAR★Methods

### Key resources table

**Table:** 

REAGENT or RESOURCE	SOURCE	IDENTIFIER
**Antibodies**		

Anti-KIF22 antibody	GeneTex	Cat#GTX66353; RRID:AB_2943604
Anti-Ki67 antibody	Abcam	Cat#ab15580; RRID:AB_443209
Anti-cleaved caspase3 antibody	Cell signaling technologies	Cat#9664; RRID:AB_2070042
Anti-α-tubulin antibody	Fujifilm Wako	Cat#017–25031; RRID:AB_2943605
Anti-Myc-tag antibody	MEDICAL & BIOLOGICAL LABORATORIES CO., LTD.	Cat#M192-3; RRID:AB_11160947
Anti-GAPDH, HRP conjugate	MEDICAL & BIOLOGICAL LABORATORIES CO., LTD.	Cat#M171-7; RRID:AB_10699462
Anti-Mouse, HRP conjugate	Jackson ImmunoResearch Laboratories	Cat#115-035-062; RRID:AB_2338504
Anti-Rabbit, HRP conjugate	Jackson ImmunoResearch Laboratories	Cat#111-035-045; RRID:AB_2337938
Alexa Fluor 546-conjugated anti-rabbit IgG	Thermo Fisher Scientific	Cat#A-11035; RRID:AB_2534093
Alexa Fluor 488-conjugated anti-rabbit IgG	Thermo Fisher Scientific	Cat#A-32731; RRID:AB_2633280
Alexa Fluor 555-conjugated anti-mouse IgG	Thermo Fisher Scientific	Cat#A-32727; RRID:AB_2633276
Alexa Fluor 488-conjugated anti-mouse IgG	Thermo Fisher Scientific	Cat#A-32723; RRID:AB_2633275

**Bacterial and virus strains**		

5-alpha Competent E. coli	New England Biolabs	Cat#C2987H

**Chemicals, peptides, and recombinant proteins**		

Alcian blue 8GX	Merck	Cat#A5268-10G
Alizarin Red S	Fujifilm wako	Cat#011-01192
Methyl-methacrylate and 2-hydroxyethyl methacrylate mixture	Fujifilm wako	Cat#086-04385
Dulbecco’s Modified Eagle Medium (DMEM)/Ham’s F12	Fujifilm Wako	Cat#048-29785
Streptomycin	Fujifilm Wako	Cat#168-23191
Insulin-Transferrin-Selenium (ITS) solution	Thermo Fisher Scientific	Cat#41400045
DMEM	Fujifilm Wako	Cat#043-30085
Collagenase	Fujifilm Wako	Cat#034-22363
TRIzol™ Reagent	Thermo Fisher Scientific	Cat#15596026
Power Up SYBR™ Green Master Mix	Thermo Fisher Scientific	Cat#A25742
ProLong™ Glass Antifade Mountant	Thermo Fisher Scientific	Cat#P36984
4′,6-diamidino-2-phenylindole, dihydrochloride (DAPI)	Dojindo	Cat#D212
Protease Inhibitor Cocktail SetⅠ	Fujifilm Wako	Cat#165-26021
PhosSTOP™	Merck	Cat#4906845001
Immobilon ECL Ultra Western HRP Substrate	Merck	Cat#WBULS0100
Nocodazol	Selleck Biotech	Cat#S2775

**Critical commercial assays**		

PrimeSTAR Max	Takara Bio	Cat#R045A
PrimeSTAR Mutagenesis Basal Kit	Takara Bio	Cat#R046A
Fast Gene™ RNA Basic Kit	Nippon Gene	Cat#FG-80050
High-Capacity cDNA Reverse Transcription Kit	Thermo Fisher Scientific	Cat#4368814
Cell Counting Kit-8 (CCK8)	Dojindo	Cat#CK04
MEBSTAIN Apoptosis TUNEL Kit Direct	MBL	Cat#8445
Dual-Glo Luciferase Assay System	Promega	Cat#E2920
Nano-Glo Dual-Luciferase Reporter Assay System	Promega	Cat#N1610

**Experimental models: Cell lines**		

ATDC5 cells	Riken Bio Resource Center Cell Bank	Cat#RCB0565; RRID:CVCL_3894
HEK293 cells	Riken Bio Resource Center Cell Bank	Cat#RCB1637; RRID:CVCL_0045

**Experimental models: Organisms/strains**		

C57BL/6N	CLEA Japan	N/A
Kif22ΔC/C57BL/6N	This paper	N/A

**Oligonucleotides**		

Primer: *Kif22* Forward: GGCCGCTGTGTAAGCAAAG	This paper	N/A
Primer: *Kif22* Reverse: TCCTTCGCTTCTGTCTCTCCA	This paper	N/A
Primer: *Col2a1* Forward: GCTGGTGAAGAAGGCAAACGAG	Nakatomi et al.^37^	https://doi.org/10.1016/j.bone.2019.01.002
Primer: *Col2a1* Reverse: CCATCTTGACCTGGGAATCCAC	Nakatomi et al.^37^	https://doi.org/10.1016/j.bone.2019.01.002
Primer: *Acan* Forward: CAGCTGCCCTTCACATGTAAA	Nakatomi et al.^37^	https://doi.org/10.1016/j.bone.2019.01.002
Primer: *Acan* Reverse: TGGACAAAGCCCTCAGTACACT	Nakatomi et al.^37^	https://doi.org/10.1016/j.bone.2019.01.002
Primer: *Tbp* Forward: GAAGCTGCGGTACAATTCCAG	Goto et al.^38^	https://doi.org/10.1177/00220345221075966
Primer: *Tbp* Reverse: CCCCTTGTACCCTTCACCAAT	Goto et al.^38^	https://doi.org/10.1177/00220345221075966
Primer: qPCR Kif22 wt check Forward: aacctggagtgattcctcggg	This paper	N/A
Primer: qPCR Kif22 ΔC check Forward: aacctggagtgattcctcAgC	This paper	N/A
Primer: qPCR Kif22 check Reverse: atgcaggatccaagaggtctaatac	This paper	N/A

**Recombinant DNA**		

pBAsi-mU6 vector	Takara Bio	Cat#3222
Cosmid vector pAxcwit2	Takara Bio	Cat#6174
pcDNA3.1	Thermo Fisher Scientific	Cat#V79020
Cosmid vector pAxEFwtit2	Takara Bio	Cat#6174
pTagRFP-tubulin	Evrogen	Cat#FP145
pGL4.74[hRluc TK]	Promega	Cat#E6921
pBiT1.1-C [TK/LgBiT] Vector	Promega	Cat#N196A
pBiT2.1-C [TK/SmBiT] Vector	Promega	Cat#N197A
pGL4.53[luc2/PGK] Vector	Promega	Cat#E501A
Col2a1-luc	Hata et al.^39^^,^^40^	

**Software and algorithms**		

ImageJ	Schneider et al.^41^	https://imagej.nih.gov/ij/

### Resources availability

#### Lead contact

Further information and requests for resources should be directed to and will be fulfilled by the lead contact, r15matsubara@fa.kyu-dent.ac.jp (Takuma Matsubara).

#### Materials availability

Kif22 plasmids and Kif22 mutant mice generated in this study will be distributed upon request.

#### Data and code availability

• Code: This paper does not report original code.
• Data: Original pictures and western blot images have been deposited at Mendeley and are publicly available as of the date of publication. The DOI is https://doi.org/10.17632/mzvmmt67hx.1.
• Any additional information required to reanalyse the data reported in this paper is available from the lead contact upon request.

### Experimental model and study participant details

#### Animals

*Kif22* mutant mice were generated by CRISPR/Cas9 genome editing (Setsuro Tech). The guide RNA sequence was 5′- aggagguccaugagagcccg -3′, complementary to genomic DNA sequence 5′- CGGGCTCTCATGGACCTCCT - 3′ starting at the codon encoding R144. The obtained mutant allele sequence with indel underlined was 5′- CAGCGCTCTCATGGACCTCCT - 3’. This mutation leads to a R144fs∗16 frameshift mutation terminating at amino acid 159 (*Kif22*^*ΔC*^). Mutation was confirmed by Sanger sequencing.

C57BL/6N wild-type mice were purchased from CLEA Japan. All mice were handled in accordance with guidelines from the Animal Care and Use Committee of Kyushu Dental University based on the Animal Research: Reporting of *In Vivo* Experiments (ARRIVE) guidelines (Approval#20-14).

#### Cell lines

ATDC5 cells (Riken Bio Resource Center Cell Bank) were maintained in Dulbecco’s Modified Eagle Medium (DMEM)/Ham’s F12 (Fujifilm Wako) supplemented with 5% Fetal Bovine Serum (FBS) and 100 units/ml penicillin G with 100 μg/mL streptomycin (Fujifilm Wako).

To generate stable cells, empty pcDNA3.1 vector, myc-tagged wild-type Kif22 plasmid and myc-tagged Kif22 P143L mutant plasmid were transfected into ATDC5 cells. After 2 days culture, stable cells were selected with 10 μg/mL G418 (Fujifilm Wako) for 7 days. Expression of KIF22 was confirmed by western blotting analysis.

HEK293 (Riken Bio Resource Center Cell Bank) cells were maintained in DMEM (Fujifilm Wako) supplemented with 10% FBS and 100 units/mL penicillin G with 100 μg/mL streptomycin.

### Method details

#### Skeletal preps

After removing skin and internal organs, 1-day-old mice were fixed with 95% ethanol. Fat was then removed with acetone after which samples were then stained with 1% Alcian blue 8GX (Merck), 20% acetic acid, 80% ethanol solution for 3 h and then dehydrated with 95% ethanol. After incubating with a 1% KOH and 50% ethanol solution, samples were stained with a solution containing 0.03% Alizarin Red S and 1% KOH for 1 h. After destaining with 1% KOH, samples were clarified with glycerol.^37^^,^^42^

#### Histomorphometric analysis

Tibia were dissected from 8-week-old wild-type (*n* = 8) and *Kif22*^*wt/ΔC*^ (*n* = 10) male mice and fixed in 70% ethanol for 3 days. Fixed tibias were dehydrated through a series of graded ethanol concentrations and then infiltrated and embedded in methyl-methacrylate and 2-hydroxyethyl methacrylate mixture (Fujifilm wako). Undecalcified 4 μm-thick microtome sections were stained with the von kossa method. Images were captured with Olympus cellSens software (Olympus) and growth plates analyzed as previously described.^43^ For histomorphometric analysis, sections were stained with 0.05% toluidine blue solution (pH 7.0). Histomorphometric analysis of growth plate thickness at the proximal tibia and trabecular bone were performed with Histometry RT camera software (System Supply). The histomorphometric parameters were described using standardized nomenclature.^44^

#### MicroCT analysis

Tibia were dissected from 8-week-old wild-type (*n* = 4) and *Kif22*^*wt/ΔC*^ (*n* = 4) male mice and fixed in 4% paraformaldehyde for 24 h and then stored in 70% ethanol. Micro-computed tomography (μCT) scanning of the tibiae was performed according to the manufacturer’s instructions using a Scanco Medical μCT35 System (SCANCO Medical) with an isotropic voxel size of 6 μm. We defined the regions of interest (ROI) as 200 slices starting 60 μm proximal to the distal growth plate in the femur. These images were used for 3D reconstruction and analysis.^45^ Structural parameters (3D) included cortical structure, and trabecular structure according to established guidelines.^46^

#### Antibodies

Anti-KIF22 antibody (GTX66353) was purchased from GeneTex. Anti-Ki67 antibody (ab15580) was from Abcam. Anti-cleaved caspase3 antibody (9664S) was from Cell signaling technologies. Anti-α-tubulin antibody (017–25031) was from Fujifilm Wako. Anti-Myc-tag antibody (M192-3) and anti-GAPDH HRP (M171-7) were from MBL. Anti-Mouse HRP (115-035-062) and Anti-Rabbit HRP (111-035-045) were from Jackson ImmunoResearch Laboratories. Alexa Fluor 546-conjugated anti-rabbit IgG (A-11035), Alexa Fluor 488-conjugated anti-rabbit IgG (A-32731), Alexa Fluor 555-conjugated anti-mouse IgG (A-32727) and Alexa Fluor 488-conjugated anti-mouse IgG (A-32723) were from Thermo Fisher Scientific.

#### Chondrocyte differentiation of ATDC5 cells

ATDC5 cells were plated at 35,000 cells/cm^2^ and culture medium was additionally supplemented with 1% (v/v) Insulin-Transferrin-Selenium (ITS) solution (Thermo Fisher Scientific).^47^ Chondrocyte matrix was stained with 1% Alcian blue stain-8GX.^37^^,^^47^

#### Primary chondrocytes

Costal cartilage was dissected from P2 mice and processed with 0.1% collagenase (Fujifilm Wako) in PBS overnight. Primary chondrocytes were collected and washed with PBS.^37^ The cells were plated at 35,000 cells/cm^2^ in DMEM/Ham’s F12 containing 5% FBS, 0.1% dexamethasone, 50 μM L-ascorbic acid 2-phosphate, 40 μg/mL L-proline, 1 mM Sodium pyruvate and 1% ITS. After 7 days, the cells were stained with Alcian blue and mRNA was harvested.

#### Plasmids

Control shRNA (Target: gcccagccacggaccttta) or *Kif22* shRNA (Target: cctgttaagctgtctcagaaa) were inserted into pBAsi-mU6 vector from Takara Bio. The shRNA and U6 promoter were subcloned into the cosmid vector pAxcwit2 from Takara Bio.^48^

Mouse *Kif22* and *Sox9* were PCR cloned from mouse articular cartilage cDNA library with PrimeSTAR Max (Takara Bio) and inserted into pcDNA3.1 (Thermo Fisher Scientific). A Myc-tag sequence was inserted at the C-terminus. Site-directed mutagenesis to obtain P143L was performed with PrimeSTAR Mutagenesis Basal Kit (Takara Bio) using a 5′- agtgattcTtcgggctctcatggacctc -3′ primer. Mutations were confirmed by Sanger sequencing (Azenta life sciences). Myc-tagged KIF22 wild-type was subcloned into the cosmid vector pAxEFwtit2 from Takara Bio.^48^ Fresh adenovirus was produced by transfecting cosmid vectors into HEK293 cells and titers determined as previously described.^48^ Col2a1-luc plasmid was kindly provided by Dr. Riko Nishimura, Osaka University Graduate School of Dentistry.^39^^,^^40^

#### Real-time quantitative PCR

Brain, Lung, Heart, Thymus, Liver, Spleen, Kidney, growth plate, cortical bone, bone marrow, muscle, and adipose tissue were collected from 5-week-old male mice. Tissues were homogenized with TRIzolⓇ Reagent (Thermo Fisher Scientific). Chloroform was then added and following centrifugation the upper aqueous supernatant containing mRNA was purified with Fast Gene RNA Basic Kit from Nippon Genetics. For ATDC5 cells, mRNA was harvested using Fast Gene RNA Basic Kit according to the manufacturer’s protocol. mRNA was reverse transcribed into cDNA using High-Capacity cDNA Reverse Transcription Kit (Thermo Fisher Scientific). qPCR was performed using Quantstudio3Ⓡ real-time PCR system from Thermo Fisher Scientific with Power Up SYBR Green Master Mix (Thermo Fisher Scientific). Primer sequences are shown in the table below. qPCR data were analyzed with the ΔΔCt method using *Tbp* as the housekeeping gene. To quantify expression across various tissues, brain tissue was used as the internal control. For allele specific qPCR, a standard curve method was used to control for primer amplification efficiency. The standard curve was generated by serial dilution of *Kif22*^*wt/ΔC*^ mouse genomic DNA. *Kif22 WT* or *ΔC* expression levels was normalized to *Tbp*.

**Table:** qPCR primers

Gene	Forward primer (5′–3′)	Reverse primer (5′–3′)
*Kif22* (Kinesin family member 22)	ggccgctgtgtaagcaaag	tccttcgcttctgtctctcca
*Col2a1* (Type Ⅱ Collagen alpha 1)	gctggtgaagaaggcaaacgag	ccatcttgacctgggaatccac
*Acan* (Aggrecan)	cagctgcccttcacatgtaaa	tggacaaagccctcagtacact
*Tbp* (TATA box binding protein)	gaagctgcggtacaattccag	ccccttgtacccttcaccaat

#### Immunostaining

Tibia were dissected from 2-week-old male mice and fixed with 4% paraformaldehyde (PFA) solution. Bones were then decalcified with 0.24 M EDTA for 1 week. Sample were dehydrated in ethanol, embedded in paraffin, and cut into 4 μm sections.^37^ Following removal of paraffin, antigen retrieved was performed with 0.01 M sodium citrate buffer at 95°C for 10 min. Next, slides were rinsed with PBS and then blocked with 5% bovine serum albumin (BSA) in PBS for 1 h. Primary antibody incubation was performed overnight at 4°C. The sections were then rinsed with PBS and incubated with secondary antibodies for 1 h at room temperature (RT). Coverslips were then mounted with ProLong Glass Antifade Mountant (Thermo Fisher Scientific).

For ATDC5 immunostaining, cells were plated at 10,000 cells/cm^2^ on a 12 mm cover glass. After cell culture, cells were fixed with 4% formaldehyde for 10 min and then permeabilized with 0.2% Triton X-100 in PBS for 15 min. Cells were then blocked with 5% BSA for 1 h and then incubated with primary antibodies in 5% BSA overnight at 4°C. Next, cells were incubated in secondary antibody containing 5% BSA/PBS for 1 h at RT. Nuclei were stained with 4′,6-diamidino-2-phenylindole, dihydrochloride (DAPI) (Dojindo) and slides mounted with ProLong Glass Antifade Mountant (Thermo Fisher Scientific). Images were acquired on a BZ-X810 microscope (Keyence).^49^

#### Western blotting

Cells were lysed with RIPA buffer (1% Triton X-100, 0.1% Sodium Dodecyl Sulfate (SDS), 1% Sodium deoxycholate, 150 mM NaCl, 10 mM Tris and 5 mM EDTA) supplemented with containing Protease Inhibitor Cocktail Set I (Fujifilm Wako) and PhosSTOP (Merck). After sonication and centrifugation at 15,000 rpm for 15 min, the supernatant was collected and boiled with sample buffer (125 mM Tris, 40% glycerol, 4% SDS, 0.2 M dithiothreitol and 0.01% bromophenol blue) for 5 min at 95°C. Samples were separated on 7.5% and 10% SDS- Polyacrylamide gel electrophoresis (PAGE) gels and transferred to polyvinylidenedifluoride (PVDF) membranes. Membranes were blocked with 5% BSA in Tris-Buffered Saline with Tween 20 (TBST) for 2 h at RT and incubated with primary antibody in 5% BSA, TBS-T at 4°C overnight. Membranes were then washed with TBS-T and incubated with secondary antibody containing 5% BSA, TBS-T. Finally, blots were imaged with a LAS4000 (Fujifilm Wako) using Immobilon ECL Ultra Western HRP Substrate (Merck).

#### Cell viability assay

ATDC5 cells were plated at 12,500 cells/cm^2^ in 96-well plates. Cell viability was assessed every 24 h with the Cell Counting Kit-8 (CCK8) from Dojindo, according to the manufacturer’s protocol.^50^

#### Tunel assay

ATDC5 cells were plated at 10,000 cells/cm^2^ on 12 mm coverslips. After fixation, apoptosis was assessed with MEBSTAIN Apoptosis TUNEL Kit Direct from MBL, according to the manufacture’s protocol.

#### Analysis of mitosis

ATDC5 cells were plated at 10,000 cells/cm^2^ on 12-mm coverslips and cultured for 24 h. Cells were then treated with 40 ng/mL nocodazole (Selleck Biotech) for 14 h and then the nocodazole removed and cells washed. After another 30 min culture,^28^^,^^51^ cells were fixed and immunostained with anti-α-tubulin and Alexa Fluor 555.

#### Luciferase reporter assays

Col2a1-luc, pcDNA3.1, Sox9, Kif22 wild-type and mutant, and pGL4.74[Rluc TK] (Promega) were transfected into ATDC5 cells using Lipofectamine 2000. After 24h cells were lysed and luciferase activity was measured using a Dual-Glo Luciferase Assay System (Promega) and an Infinite 200 plate reader (TECAN). Firefly luciferase activity was normalized to Renilla luciferase.

#### Nanobit protein-protein interaction analysis

*Sox9* were subcloned into pBiT1.1-C [TK/LgBiT] (Promega) to generate SOX9-Lgbit fusion protein construct. Wild-type *Kif22* and *Kif22* mutants were subcloned into pBiT2.1-C [TK/Smbit] Vector (Promega) to generate the KIF22-SmBit fusion protein constructs. Lgbit and Smbit fusion plasmids were transfected into ATDC5 cells using Lipofectamine 2000 and cultured for 24 h. pGL4.53[luc2/PGK] (Promega) was co-transfected as an internal transfection efficiency control. Interaction of Lgbit and Smbit fusion proteins results in formation of a functional Lgbit-Smbit Nanoluc luciferase protein. Luciferase activity measured using the Nano-Glo Dual-Luciferase Reporter Assay System (Promega) and an Infinite 200 plate reader (Tecan). Nanoluc activity was normalized with Luc2 activity.

### Quantification and statistical analysis

Statistical significance between means was analyzed using Student’s t test for 2 groups comparison or one-way ANOVA followed by a Tukey-Kramer post-hoc test for multi groups comparison. Statistical significance was threshold was set at *p* < 0.05. Data are expressed as mean ± standard deviation (SD). All *in vitro* experiments were independently performed at least 3 times and similar results were obtained.

## Supplemental information

Document S1. Figures S1‒S6

Table S1. Statical data of the graph at Figure 1 to 6, related to Figure 1, 2, 3, 4, 5, and 6
