# Supplementary material for: KIF22 regulates mitosis and proliferation of chondrocyte cells
Source: iScience. 2024 May 31;27(7):110151. doi: 10.1016/j.isci.2024.110151 (PMC11233920; doi:10.1016/j.isci.2024.110151)
Supplement: Document S1. Figures S1‒S6 [file mmc1.pdf]

iScience, Volume *27*

Supplemental information

KIF22 regulates mitosis and proliferation of chondrocyte cells

Hiroka Kawaue, Takuma Matsubara, Kenichi Nagano, Aoi Ikedo, Thira Rojasawasthien, Anna Yoshimura, Chihiro Nakatomi, Yuuki Imai, Yoshimitsu Kakuta, William N. Addison, and Shoichiro Kokabu

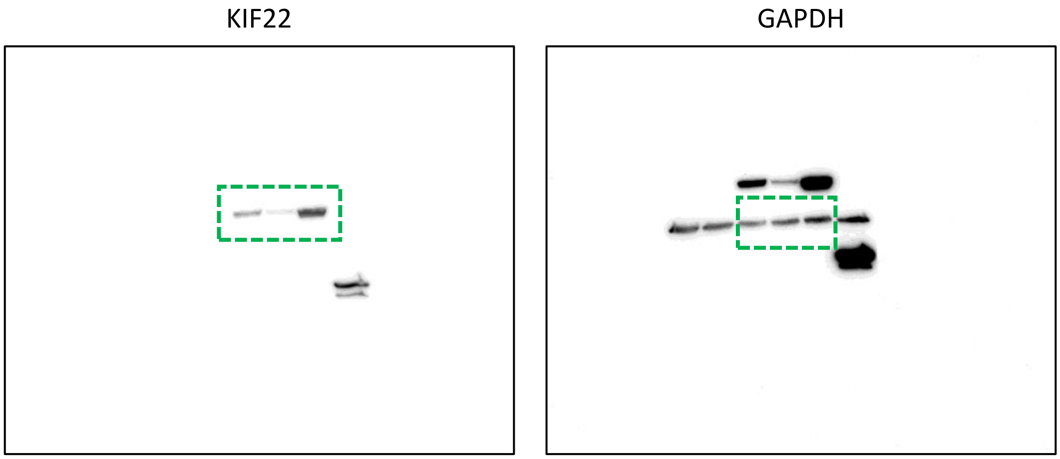

Supplemental Figure 1. Uncropped image of Figure 2A, related to Figure 2A.
Uncropped image of Figure 2A. The area enclosed by the dashed line is shown in Figure 2. After KIF22 was blotted first, GAPDH was reblotted at same membrane.

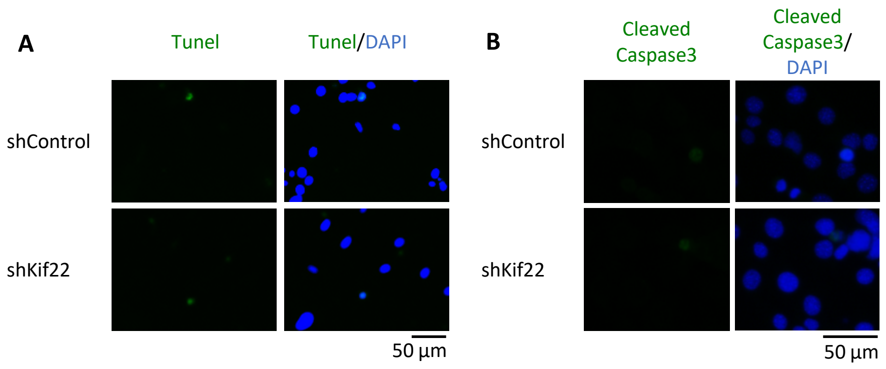

Supplemental Figure 2. KIF22 knockdown does not affect apoptosis, related to Figure 2.
(A) Tunel staining and (B) anti-cleaved Caspase 3 immunostaining of ATDC5 cells 48 h after infection with control (shControl) or *Kif22*-targeting shRNA (shKif22). Nuclei are stained with DAPI. Scale bar = 50 µm

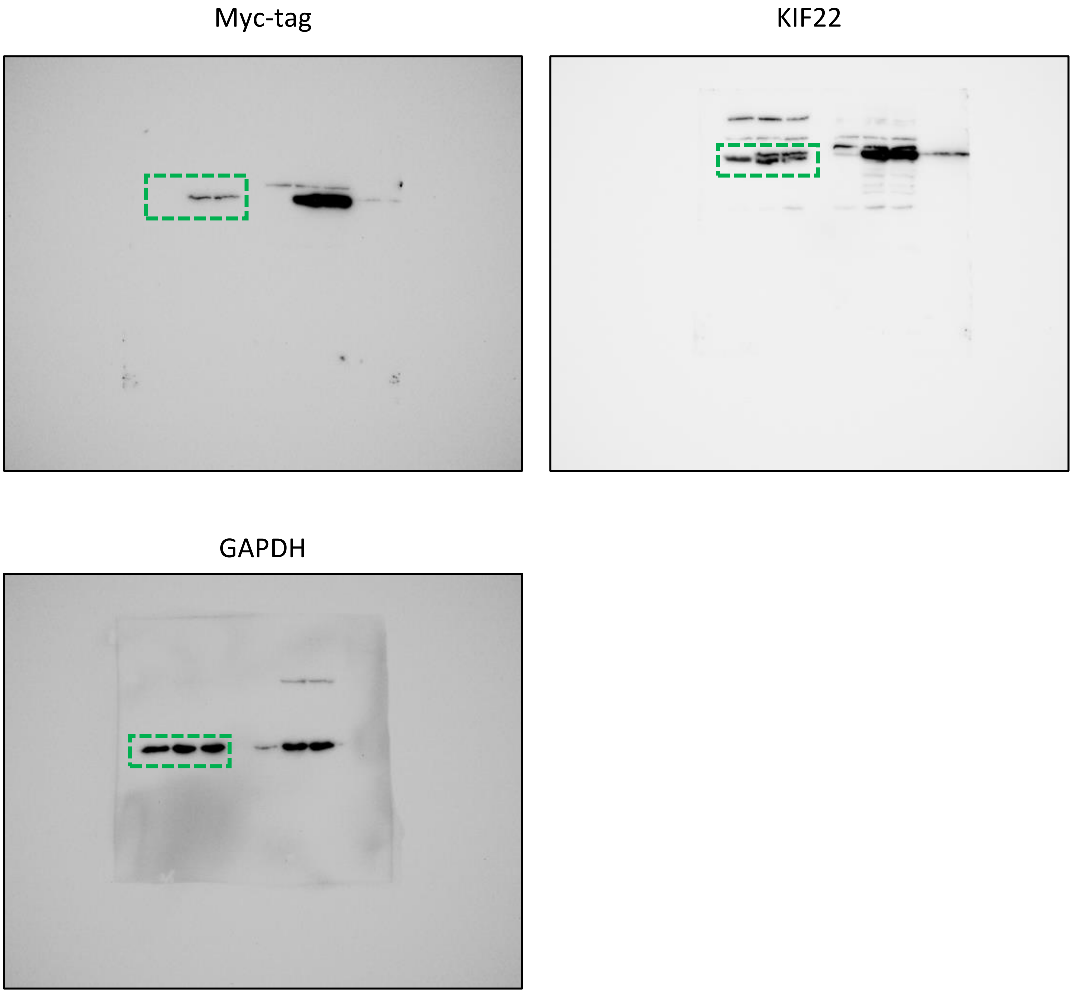

Supplemental Figure 3. Uncropped image of Figure 5A, related to Figure 5A.
Uncropped image of Figure 5A. The area enclosed by the dashed line is shown in Figure 2. KIF22 was blotted first, Myc-tag was reblotted 2nd, and finally GAPDH was reblotted at same membrane.

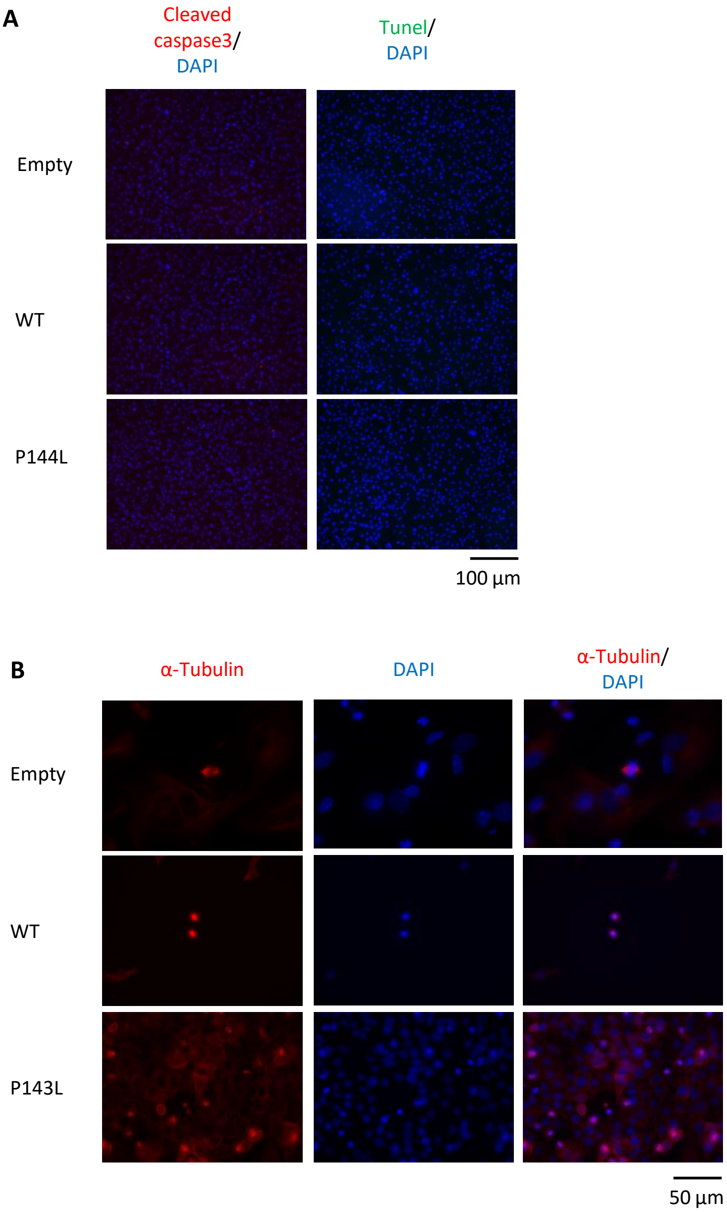

Supplemental Figure 4. Kif22 R143L disturbs mitosis, but does not affect apoptosis, related to Figure 5.

(A) Apoptosis was examined by immunostaining of cleaved caspase 3 (left panel) or tunel staining (right panel). There were no significance between empty vector transfected KIF22 cells, KIF22 wild type cells and KIF22 P143L cells.

(B) Pictures of the field of view containing Fig. 5E were taken with a 40x objective lens.

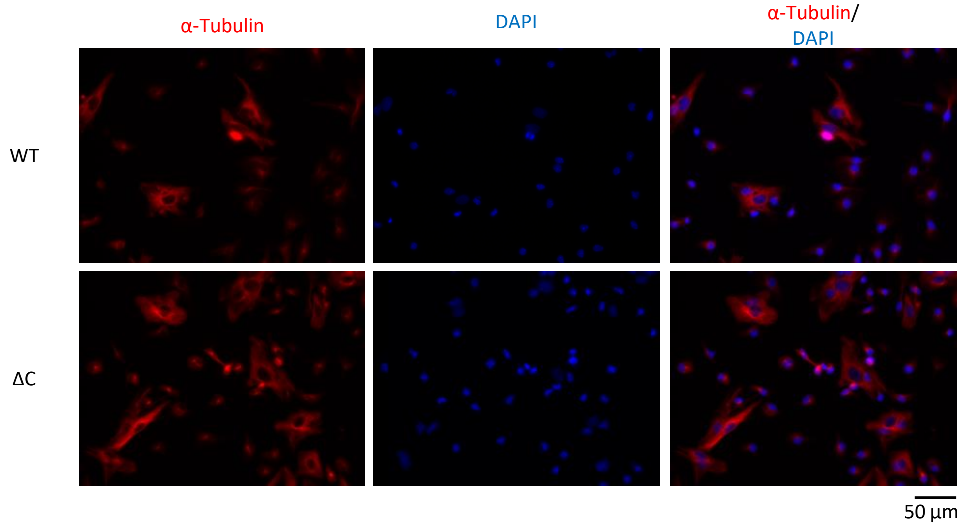

Supplemental Figure 5. Lower magnification image of Fig. 6E, related to Figure 6.

Pictures of the field of view containing Fig. 6E were taken with a 40x objective lens.

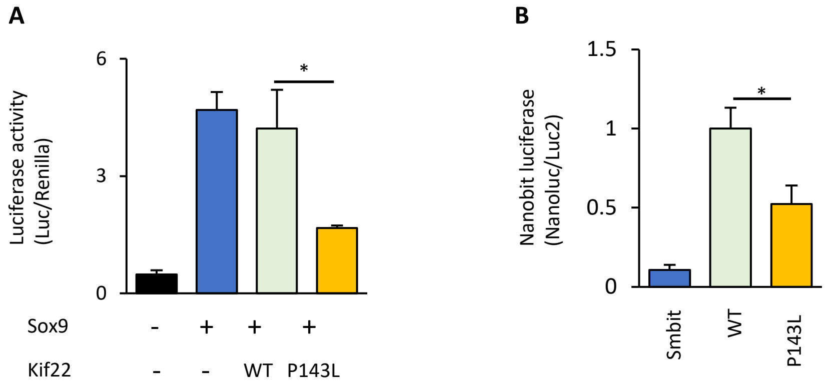

Supplemental Figure 6. Kif22 mutants weakly bind to Sox9 and reduce Sox9 transcription activity, related to Figure 5.
(A) Col2a1-luc, Sox9, pGL4.74[Rluc TK], wild-type KIF22 (WT), and KIF22 P143L (P143L) are transfected into ATDC5 cells. After 1d culture, luciferase activity was measured and normalized by Renilla activity (*; $p < 0.05$, $n = 4$). (B) Sox9-Lgbit, pGL4.53[luc2/PGK], and indicated Smbit-tagged Kif22 constructs are transfected into ATDC5 cells. After 2 d culture, Nanoluc activity was measured as binding affinity and normalized by luciferase activity (*; $p < 0.05$, $n = 4$).
