# Supplementary material for: KIF22 regulates mitosis and proliferation of chondrocyte cells
Source: iScience. 2024 May 31;27(7):110151. doi: 10.1016/j.isci.2024.110151 (PMC11233920; doi:10.1016/j.isci.2024.110151)
Supplement: Table S1. Statical data of the graph at Figure 1 to 6, related to Figure 1, 2, 3, 4, 5, and 6 [file mmc2.docx]

**Supplemental table. Statical data of the graph at Figure 1 to 6, related to Figure 1, 2, 3, 4, 5, and 6**
